# Mamba-BEV: A Multiscale State-Space Framework for 3D Object Detection from Point Clouds

**DOI:** 10.3390/s26165271

**Published:** 2026-08-20

**Authors:** Yuyang Liu, Jiabin Wang, Min Mao, Kun Zhang, Yu Xu, Mingchen Zhu, Xianjun Wu

**Affiliations:** 1 College of Physics and Electronic Engineering, Xinyang Normal University, Xinyang 464000, China; wmushan952@163.com (J.W.);; 2 School of Information and Communication Engineering, University of Electronic Science and Technology of China, Chengdu 611731, China; 3 Xinyang Gumai Optronics Co., Ltd., Xinyang 464000, China; 4 School of Geomatics Science and Technology, Nanjing Tech University, Nanjing 210009, China; 5 Xinyang Central Semiconductor Technology Co., Ltd., Xinyang 464000, China

**Keywords:** 3D object detection, point cloud, state-space model, Mamba, bird’s-eye view (BEV)

## Abstract

LiDAR point clouds are sparse, irregular, and unevenly distributed, which makes representative feature extraction challenging for 3D object detection. Currently, Mamba modules have been increasingly applied to 3D object detection due to their ability to efficiently model global spatial dependencies. However, preserving geometric structures while modeling global spatial dependencies remains challenging in Mamba-based frameworks. In view of this, this paper proposes a single-stage 3D object detection framework for LiDAR-based point clouds. First, this paper designs a hierarchical multiscale structure called Multiscale Voxel–Point Alternating Fusion (MVPF) Module. Within this module, the 2DMamba module is introduced into the feature extraction stage at each scale to model global spatial dependencies in the BEV plane through selective state-space scanning. Second, we design a Z-to-Channel cross-dimensional reorganization strategy that merges the voxel Z dimension into the channel dimension, yielding a BEV-form feature representation suitable for 2DMamba processing. Third, this paper proposes a Local Voxel Feature Enhancement (LVFE) module composed of a Point-to-Voxel Feature Aggregation (PVFA) module, a Voxel Densification Module (VDM), and a coordinate-indexed Voxel-to-Point Mapping (VPM) module, which enhances local voxel representations while maintaining point–voxel spatial correspondence. On the KITTI validation set, Mamba-BEV achieved higher detection accuracy and inference speed than the baseline. Under the Moderate difficulty level of AP_R11_, the 3D AP for Car, Pedestrian, and Cyclist improved by 0.6%, 1.5%, and 0.4%, respectively, compared to the baseline, while the inference speed increased from 44.05 FPS to 67.11 FPS. These results demonstrate the effectiveness of the proposed Mamba-BEV for point cloud-based 3D object detection.

## 1. Introduction

LiDAR-based point cloud 3D object detection is a fundamental perception task in autonomous driving, intelligent robotics, and intelligent transportation systems, aiming to accurately perceive the position, size, and class of surrounding objects in complex environments. As an active 3D perception sensor, LiDAR can capture precise geometric coordinates, reflectance intensity, and spatial structural information, which could provide a reliable data foundation for object localization and recognition in complex outdoor scenes. However, it is still a vital issue for distant, small as well as partially occluded objects detection due to the sparsity, irregularity, and nonuniform density of point clouds. These characteristics make it challenging for detection models to simultaneously achieve fine-grained geometric information preservation, global spatial dependency exploration, and real-time inference deployment.

Existing 3D object detection methods based on point clouds can generally be categorized into point-based methods, voxel-based methods, and hybrid point-voxel methods. Point-based methods directly process raw point sets and are effective at preserving fine-grained geometric structures, e.g., PointNet [1], PointNet++ [2], PointRCNN [3], and 3DSSD [4]. However, most of these kinds of methods face high computational costs, especially in large-scale outdoor scenes. In order to decrease the computation complexity, voxel-based methods were proposed in the literature. Voxel-based methods partition irregular point clouds into regular voxel grids and use sparse convolution for feature extraction, such as VoxelNet [5], SECOND [6], and Voxel–RCNN [7]. However, the voxelization process may introduce quantization errors, leading to the loss of fine-grained geometric information. To address this limitation, methods such as PV-RCNN [8] and HVPR [9] combine point-level representations with voxel-level representations. These approaches preserve the local geometric details of the point cloud while utilizing voxel features to model spatial structural information, thereby demonstrating strong object recognition capabilities across various scenarios. However, such methods typically require complex point-to-voxel mappings and multi-branch feature fusion, which can easily introduce additional computational overhead.

Recently, Transformer-based methods have been widely adopted in 3D object detection to enhance the modeling of global spatial dependencies. Among them, VoxSeT [10] was proposed to reduce the computational complexity of full self-attention through a voxel set attention module, which could achieve good performance on the KITTI [11] dataset. However, VoxSeT still has several limitations. First, voxel set attention mainly focuses on local or set-level feature interactions, which makes it difficult to fully model global relationships among distant voxels. Second, local voxel feature modeling cannot well capture fine-grained geometric features in sparse point clouds. Third, multiscale voxel feature aggregation may introduce spatial misalignment during downsampling and upsampling, thereby reducing the consistency of cross-scale geometric representations. To address this, Mamba is widely used to model global spatial dependency with linear computational complexity, such as for large-scale BEV (Bird’s-Eye View) features. Given this, a growing number of studies have applied Mamba to point cloud-based 3D object detection [12,13,14]. However, the original 2DMamba [15] was designed primarily for images, which could not extract BEV feature of sparse point cloud effectively. Therefore, an effective BEV state-space model should not only model global spatial relationships within the BEV feature domain but also maintain local geometric representations and cross-scale feature consistency.

To address the above issues, we propose Mamba-BEV, a multiscale state-space framework for 3D object detection from point clouds. This method introduces a 2DMamba module into the BEV backbone to achieve efficient global spatial feature representation. For voxel-to-BEV feature transformation, we design a Z-to-Channel cross-dimensional reorganization strategy that merges the information in the Z dimension into the channel dimension. Additionally, we propose a Local Voxel Feature Enhancement (LVFE) module, which could improve local voxel representations and preserve point–voxel spatial correspondence. Furthermore, we construct a Multiscale Voxel-Point Alternating Fusion (MVPF) model to maintain geometric consistency across different scales. Experimental results show that the proposed framework could achieve considerable performance on the KITTI dataset.

The main contributions of this paper are summarized as follows:

1. We propose Mamba-BEV, a 3D object detection framework for LiDAR point clouds. The framework introduces 2DMamba into multiscale BEV feature extraction to model global spatial dependencies while preserving the inherent 2D structure of the BEV plane. Its effectiveness is evaluated through quantitative comparisons and ablation studies on the KITTI validation set.
2. We design a Z-to-Channel cross-dimensional reorganization strategy that merges the Z and channel dimensions to directly transform voxel features into a BEV-compatible representation. Subsequently, its symmetric inverse operation, Channel-to-Z, restores the processed BEV features to the voxel representation to support subsequent voxel-to-point feature re-injection.
3. We propose a Local Voxel Feature Enhancement (LVFE) module composed of a Point-to-Voxel Feature Aggregation (PVFA) module, a lightweight Voxel Densification Module (VDM), and a coordinate-indexed Voxel-to-Point Mapping (VPM) module. LVFE enhances local voxel representations while maintaining the spatial correspondence between points and voxels, which could reduce feature mixing and spatial misalignment in the voxel-to-point feature re-injection process.
4. We develop a Multiscale Voxel-Point Alternating Fusion (MVPF) module. Within this module, point features are propagated across different voxel scales, which could maintain cross-scale geometric consistency.

## 2. Related Work

### 2.1. Voxel- and Point–Voxel Fusion-Based 3D Object Detection

Voxel-based approaches discretize unstructured sparse point clouds into regular three-dimensional voxel grids, which facilitates efficient feature extraction with convolutional neural networks. VoxelNet [5] introduced a voxel feature encoding layer to aggregate point features within each voxel and employed 3D convolutions for spatial feature extraction. However, its dense 3D convolutions incur redundant computation over a large number of empty voxels. SECOND [6] alleviated this problem by introducing sparse convolutions that operate only on non-empty voxels, significantly improving feature extraction efficiency. PointPillars [16] further divided point clouds into vertical pillars and adopted a 2D convolutional backbone to achieve efficient single-stage detection. Subsequently, Voxel R-CNN [7] further proposed a voxel RoI pooling mechanism for direct candidate feature extraction from voxel grids, enhancing localization accuracy while sustaining high inference efficiency. In contrast, point-based approaches operate directly on raw point clouds, enabling superior retention of accurate spatial coordinates and subtle geometric details. Representative paradigms such as PointNet [1], PointNet++ [2], PointRCNN [3], 3DSSD [4], and IA-SSD [17], have achieved successive improvements in point cloud feature representation learning, region proposal generation and point downsampling strategies. Although these two categories of methods have their respective advantages, they also exhibit different limitations. Voxel-based methods are computationally efficient, yet voxelization introduces quantization errors and degrades fine-grained point-level geometric information. Point-based methods preserve geometric details more effectively, but their computational cost increases significantly with the scale of the point cloud.

To combine the computational efficiency of voxel representations with the geometric precision of point representations, researchers have further developed point–voxel fusion methods. PV-RCNN [8] aggregates multiscale voxel features onto keypoints and combines them with RoI-grid pooling, while PV-RCNN++ [18] further improves local point–voxel feature representation. Fast Point R-CNN [19] and STD [20] likewise utilize voxel features for proposal generation and point-wise features for subtle refinement, yielding a balance between detection performance and computational cost. However, many point–voxel hybrid methods rely on keypoint sampling and interpolation-based feature propagation, which may introduce feature mixing or spatial misalignment during point–voxel interaction. In addition, feature propagation across different voxel scales may compromise spatial correspondence during multiscale point–voxel interaction.

To address these issues, we design a Local Voxel Feature Enhancement (LVFE) module that integrates point-to-voxel feature aggregation, lightweight voxel-neighborhood enhancement, and coordinate-indexed voxel-to-point mapping. LVFE enhances local voxel representations and directly re-injects the enhanced voxel features into their corresponding original points using the recorded point–voxel mapping, thereby reducing interpolation-induced feature mixing and preserving point–voxel spatial correspondence. Moreover, the proposed Multiscale Voxel–Point Alternating Fusion (MVPF) module regenerates voxel indices from the original point coordinates at each scale and propagates point features across different voxel scales, thereby maintaining spatial correspondence across scales.

### 2.2. 3D Object Detection with Transformers

In recent years, Transformers [21] have been introduced into 3D object detection to address the limited capability of convolutional networks in global feature modeling. The attention mechanism captures long-range relationships between features, thereby enhancing global point cloud representations. Point Transformer [22] applied the Transformer architecture to point cloud classification, demonstrating the effectiveness of attention mechanisms for 3D geometric modeling. Subsequently, several studies extended Transformers to 3D object detection, leading to representative methods [23,24,25] that employ Transformer architectures for point feature extraction, voxel feature modeling, and proposal feature enhancement.

For sparse point-cloud scenarios, DSVT [26] introduces dynamic sparse window attention to improve voxel-feature modeling efficiency. VoTr [27] combines local attention with expansion attention to enlarge the effective receptive field, while CT3D [28] employs a channel-based Transformer within RoIs to enhance bounding-box refinement through intra-region feature interactions.

Overall, Transformer-based methods have improved the contextual modeling capability of 3D point cloud detectors. However, these performance gains often come at the cost of substantial computation and GPU memory consumption. Although strategies such as local-window attention and set-based attention can partially reduce the computational burden, autonomous-driving point clouds typically contain a large number of points. Therefore, efficiently modeling global spatial dependencies while maintaining fast inference remains a key challenge in 3D object detection.

### 2.3. State-Space Models and Mamba-Based 3D Object Detection

In recent years, state-space models (SSMs) have shown strong potential for long-sequence modeling. Compared with self-attention, SSMs capture global dependencies through recurrent state updates with linear computational complexity. S4 [29] introduced structured state-space models into deep sequence modeling, providing an efficient framework for sequence representation learning. Mamba [30] further incorporated an input-dependent selective mechanism and a hardware-aware parallel scan, improving content-adaptive modeling capability.

Inspired by Mamba’s ability to model global spatial dependencies, recent studies have extended it to 3D point cloud perception. Voxel Mamba [31] combines state-space modeling with a group-free strategy to alleviate the disruption of spatial adjacency during voxel serialization. UniMamba [32] integrates the local geometric modeling capability of 3D convolutions with the global sequence modeling capability of SSMs within a unified point cloud framework. LION [33] adopts a window-based linear group RNN framework and incorporates Mamba to strengthen global spatial dependency modeling for sparse voxel features. To mitigate spatial fragmentation caused by fixed-window serialization, WinMamba [34] introduces window-shift fusion and adaptive window fusion to enhance cross-window information exchange and improve detection performance.

Despite recent progress, adapting Mamba to sparse 3D perception remains challenging. Existing 3D Mamba methods typically organize point or voxel features into sequential, grouped, or window-based representations, which may compromise the inherent 2D spatial structure of BEV features. In addition, global state-space modeling alone may be insufficient to capture local geometric features.

To address these issues, we design a Z-to-Channel strategy that transforms voxel features into a BEV-compatible representation. The transformed features are then processed by 2DMamba, which preserves the 2D spatial structure of the BEV plane and models global spatial dependencies. In addition, LVFE enhances local voxel features to compensate for the limited local feature modeling capability of 2DMamba.

## 3. Methods

**Figure 1:**
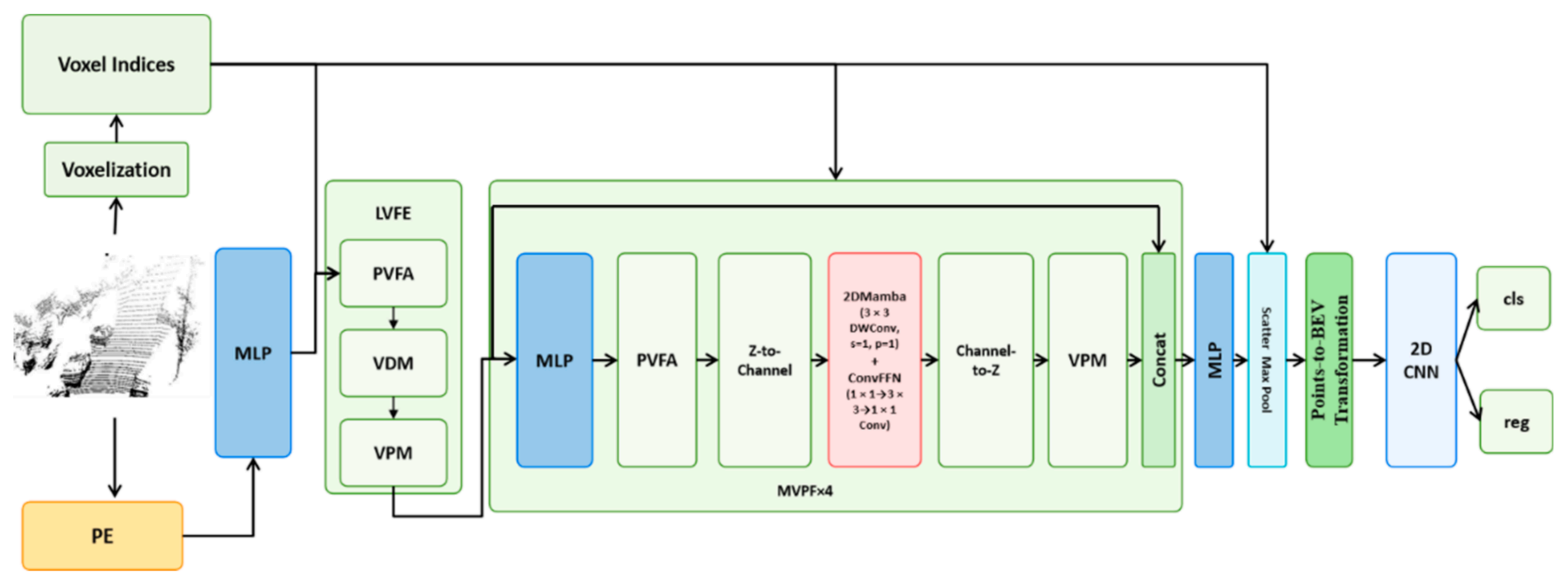
Overall network architecture of Mamba-BEV.

We propose the Mamba-BEV framework, which is designed to improve geometric information preservation, global spatial dependency modeling, and cross-scale feature consistency in point cloud-based BEV 3D detection. As shown in Figure 1, Mamba-BEV adopts an alternating point–voxel modeling framework. The model first processes the input point features using the proposed Local Voxel Feature Enhancement (LVFE) module. Within LVFE, Point-to-Voxel Feature Aggregation (PVFA) first converts the raw point features into sparse voxel representations; VDM then enhances the local representation capability of the sparse voxel features; finally, VPM re-injects the enhanced voxel features into their corresponding original points through coordinate indexing. Based on the enhanced point-level features, the model re-aggregates voxel features at different scales and performs Z-to-Channel cross-dimensional reorganization by merging the height dimension into the channel dimension, thereby generating a BEV-form feature representation. Subsequently, 2DMamba models global spatial dependencies within the BEV plane. Finally, during the multiscale alternating fusion stage, point features are propagated across different voxel scales to maintain cross-scale geometric consistency.

### 3.1. Local Voxel Feature Enhancement (LVFE)

In voxel-based point cloud processing, initial voxel features are typically obtained through an intra-voxel mean aggregation operation. Although scatter-mean is computationally efficient, it compresses all point features within the same voxel into a single feature vector. Consequently, the resulting voxel representations lack neighborhood continuity, making it difficult to effectively capture local geometric variations. Moreover, when voxel features are propagated back to the point domain, conventional interpolation-based mapping strategies may introduce cross-voxel feature mixing and spatial misalignment. These issues are particularly pronounced for sparse and small objects.

**Figure 2:**
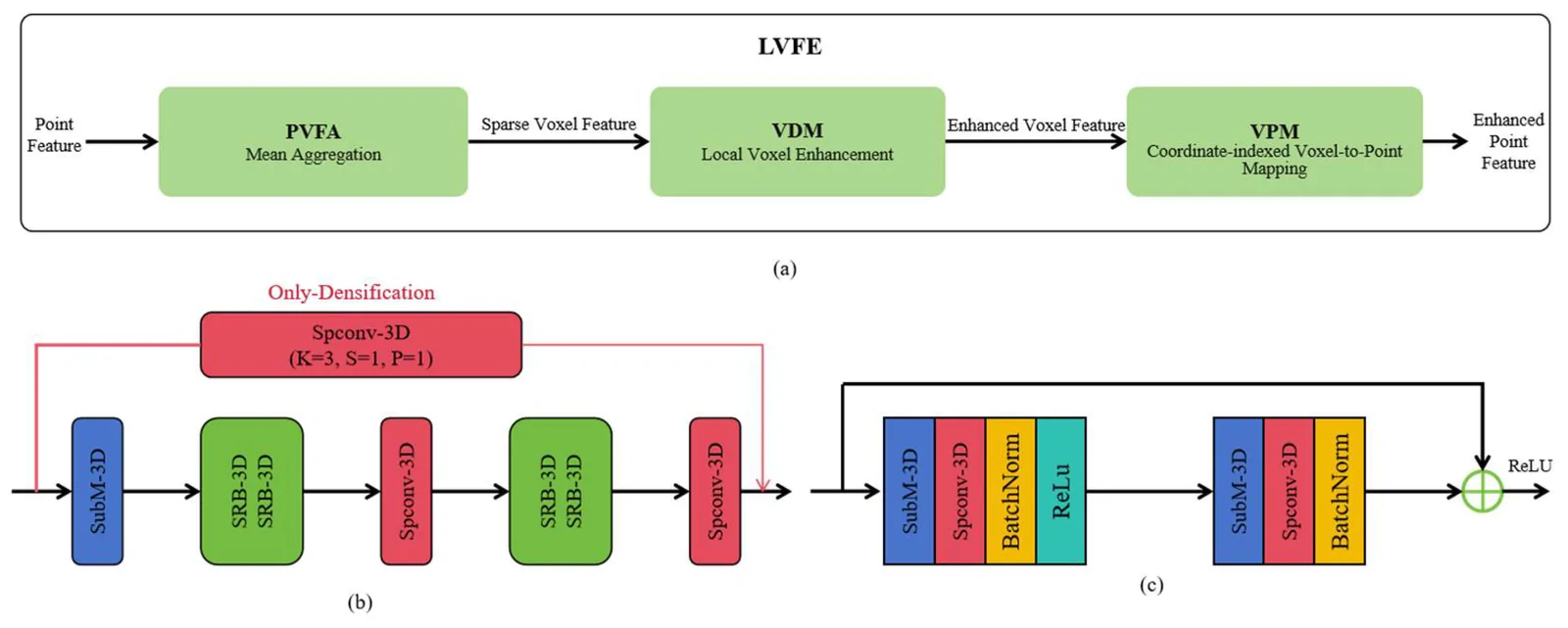
Architecture of the proposed Local Voxel Feature Enhancement (LVFE) module: (**a**) the overall LVFE pipeline; (**b**) the Voxel Densification Module (VDM); and (**c**) the SRB-3D module.

In response to the above issues, we propose a Local Voxel Feature Enhancement (LVFE) module, which enhances local voxel features while preserving point–voxel spatial correspondence. As shown in Figure 2a, LVFE consists of a Point-to-Voxel Feature Aggregation (PVFA) module, a Voxel Densification Module (VDM), and a coordinate-indexed Voxel-to-Point Mapping (VPM) module. Specifically, PVFA first aggregates the input point features within each voxel to construct initial sparse voxel representations. Subsequently, VDM aggregates information from neighboring voxels to enhance the initial voxel features and alleviate insufficient local continuity. Finally, VPM utilizes the point–voxel correspondences recorded during voxelization to re-inject the enhanced voxel features into their corresponding original points. Through this voxel feature enhancement and point-domain recovery process, LVFE helps improve local feature representations and alleviate feature mixing and spatial misalignment during voxel-to-point feature propagation.

#### 3.1.1. Point-to-Voxel Feature Aggregation (PVFA) Module

PVFA groups points into non-empty voxels according to their spatial coordinates and the predefined voxel size. The feature of each voxel is then computed by mean aggregation:

(1) $$g_{j}=\frac{1}{|P_{j}|}\sum_{p_{i}\in P_{j}}f_{i},$$

where $P_{j}$ denotes the set of points falling into the j-th voxel, $|P_{j}|$ denotes the number of points within that voxel, $f_{i}$ is the feature of point $p_{i}$, and $g_{j}$ is the aggregated voxel feature. During voxelization, PVFA also records the unique voxel coordinates and the inverse mapping index between each original point and its corresponding voxel. The resulting sparse voxel features are subsequently fed into VDM, while the ${inverse}_{i}$ mapping is retained for the following VPM module.

#### 3.1.2. Voxel Densification Module (VDM)

Within LVFE, PVFA efficiently aggregates the point features within each non-empty voxel. However, because this aggregation is performed independently within individual voxels, the resulting voxel representations lack neighborhood continuity. Consequently, these voxel features struggle to capture local geometric variations. To alleviate this issue, we introduce the Only-Densification branch of the VDM [35] prior to voxel-to-point feature re-injection. VDM aggregates information from spatially adjacent voxels through lightweight 3D sparse convolution, thereby enhancing the local continuity of sparse voxel features with limited computational overhead. The architecture of the lightweight VDM is illustrated in Figure 2b, while the SRB-3D architecture is shown in Figure 2c. The enhanced voxel features $F_{v}^{\prime }$ can be formulated as

(2) $$F_{v}^{\prime }=SpConv3D\left(F_{v}\right),$$

where $F_{v}$ denotes the initial sparse voxel features, and SpConv3D(⋅) denotes a 3D sparse convolution with a kernel size of k=3, a stride of s=1, and a padding size of p=1. Since the stride is set to 1, both the spatial resolution and coordinate layout of the sparse voxel grid remain unchanged. Therefore, each voxel can incorporate information from its local 3D neighborhood without additional downsampling.

Compared with directly stacking deeper sparse convolutional modules, the lightweight VDM is not intended to construct a complex 3D feature extraction backbone. Instead, it compensates for insufficient local neighborhood interactions while preserving the original sparse geometric structure. Therefore, it is better suited as a local voxel feature enhancement module that provides enhanced voxel features for the subsequent VPM module.

#### 3.1.3. Voxel-to-Point Mapping (VPM) Module

After VDM enhancement, the sparse voxel features have incorporated local 3D neighborhood context. These enhanced voxel features then need to be re-injected into their corresponding original points for subsequent point-level processing. Conventional interpolation-based mapping strategies are simple to implement; however, when re-injecting enhanced voxel features into the original points, they may introduce feature blurring and spatial misalignment, particularly when neighboring voxels contain points with different spatial distributions. To maintain accurate point-to-voxel correspondence during feature re-injection, we propose the Voxel-to-Point Mapping (VPM) module. This module utilizes the point-voxel correspondence recorded during the voxelization stage to re-inject the enhanced voxel features into the original points via coordinate indexing.

Specifically, the VDM output includes the enhanced features of non-empty voxels $F_{v}^{\prime }$ and their corresponding sparse voxel coordinates. To preserve the coordinate-indexed correspondence of sparse voxel features and enable accurate voxel-to-point feature retrieval, we first construct a five-dimensional dense temporary tensor:

(3) $$T\in R^{B\times H\times W\times Z\times C},$$

Here, B denotes the batch size; H and W denote the voxel-grid dimensions along the two spatial axes of the BEV plane; Z denotes the voxel-grid dimension along the vertical axis; C denotes the number of feature channels; and M denotes the number of non-empty voxels. ${(b}_{m},x_{m},y_{m},z_{m})$ represents the batch index and spatial coordinates of the m-th non-empty voxel, while “:” denotes the selection of all feature channels at the specified position. Subsequently, based on the sparse voxel coordinates output by VDM, the enhanced feature of each non-empty voxel is written to the corresponding position in tensor T, as follows:

(4) $$T\left[b_{m},x_{m},y_{m},z_{m},:\right]=F_{v}^{\prime }\left[m,:\right],\;\;\;\;\;\;\;\;\;m\;=\;1,\;2,\;\ldots ,\;M$$

Through this operation, the sparse enhanced voxel features are restored to a regular five-dimensional voxel space while preserving their 3D spatial index relationships. During the voxelization stage, the model simultaneously records the inverse mapping relationship between each original point and its corresponding unique voxel. Let j denote the set of unique voxel coordinates; then, the corresponding enhanced voxel features can be retrieved from the five-dimensional temporary tensor based on this coordinate set:

(5) $$F_{upd}[j,:]=T\;[b_{j},x_{j},y_{j},z_{j},:],$$

where $\left(b_{j},x_{j},y_{j},z_{j}\right)\;$ represents the coordinates of the j-th unique voxel. Subsequently, the unique voxel features are directly distributed to each original point using the point-to-voxel reverse index ${inverse}_{i}$:

(6) $$f_{i}^{v}=F_{upd}\left[{inverse}_{i},:\right],$$

where $\;{inverse}_{i}$ denotes the unique voxel index corresponding to the i-th original point, and $f_{i}^{v}$ denotes the enhanced voxel context features re-injected to point $P_{i}$. For different voxel scales, the model re-establishes the point-voxel correspondence based on the current scale and uses the corresponding reverse index to complete the re-injection of voxel features within that scale.

Through this process, VPM enables the enhanced voxel features to remain spatially aligned with the original point coordinates. Compared with interpolation-based voxel-to-point feature propagation methods, VPM directly assigns features according to the recorded point–voxel mapping relationship, thereby enabling direct voxel-to-point feature transfer without interpolation. Consequently, VPM re-injects the enhanced voxel features into their corresponding original points, enabling LVFE to enhance local voxel representations while maintaining the spatial correspondence between points and voxels.

### 3.2. Z-to-Channel Cross-Dimensional Reorganization and 2DMamba Global Scanning

#### 3.2.1. Z-to-Channel Cross-Dimensional Reorganization

Although LVFE enhances local voxel representations through VDM and re-injects the enhanced voxel features into the original points through VPM, 3D object detection still requires modeling spatial dependencies over a larger spatial range. The original Mamba is designed for one-dimensional sequence modeling, and directly flattening BEV features into a one-dimensional sequence may disrupt their inherent 2D spatial neighborhood structure. Therefore, to adapt voxel features to 2D state-space modeling while maintaining the spatial structure of the BEV plane, we propose a Z-to-Channel cross-dimensional reorganization strategy and combine it with 2DMamba for global spatial modeling of BEV features. After 2DMamba processing, the symmetric Channel-to-Z operation restores the BEV features to the voxel representation, enabling subsequent voxel-to-point feature re-injection through VPM.

At each scale, the current point features are first converted into voxel features through the PVFA module. Subsequently, all non-empty voxel features are placed into a regular five-dimensional voxel tensor according to their corresponding voxel coordinates:

(7) $$G\in R^{B\times H\times W\times Z\times C},$$

Here, B represents the batch size, H and W denote the voxel-grid dimensions along the two spatial axes of the BEV plane, Z denotes the voxel-grid dimension along the vertical axis, and C represents the number of channels per voxel. To construct a BEV-form representation suitable for 2DMamba processing, we merge the Z dimension into the channel dimension to obtain a four-dimensional feature tensor:

(8) $$X_{bev}=\mathrm{reshape}(G)\in R^{B\times H\times W\times (ZC)},$$

After the above transformation, the resulting feature tensor $X_{bev}$ preserves the spatial layout of the BEV plane and can be directly fed into the 2DMamba block for processing.

#### 3.2.2. 2DMamba Module

After Z-to-Channel cross-dimensional reorganization, BEV features are fed into the 2DMamba block to model global spatial dependencies within the BEV plane. Let the input features be:

(9) $$X_{bev}\in R^{B\times H\times W\times C_{bev}},$$

where B denotes the batch size, H and W denote the BEV plane grid sizes, and $C_{bev}$ denotes the channel dimension after Z-to-Channel reorganization.

The 2DMamba blocks we use follow a 2D selective scanning mechanism. Before the linear projection, RMSNorm is applied to the input BEV features in a pre-normalization manner. The normalized features are then divided into the main branch and the gated branch via linear projection:

(10) $$[x,z]={Linear}_{in}({RMSNorm(X}_{bev})),$$

where x is used for subsequent selective state-space modeling, and z is used for gated modulation. Subsequently, the main branch is then processed by a 3 × 3 depthwise convolution with stride 1 and padding 1, followed by a nonlinear activation that introduces local spatial inductive bias:

(11) $$\tilde{x}=SiLU\left({DWConv}_{2D}\left(x\right)\right),$$

Building on this, 2DMamba dynamically generates selective state-space parameters based on the input features and performs cascaded selective scans in both the horizontal and vertical directions on the 2D BEV grid. Specifically, the module first performs an intra-row selective scan along the horizontal direction of the BEV feature map to aggregate lateral spatial context; it then uses the intermediate state obtained from the horizontal scan as input to continue performing an intra-column selective scan along the vertical direction, thereby forming 2D context propagation along a horizontal–vertical continuous path. Unlike directly flattening BEV features into a one-dimensional sequence, this mechanism preserves the 2D spatial adjacency relationships of BEV features during the scanning process, thereby mitigating the disruption of spatial adjacency relationships caused by one-dimensional serialization. The spatially enhanced features obtained from the scanning process are further fused with gated branches and restored to the target channel dimension via an output projection:

(12) $$Y={Linear}_{out}\left(Gate\left(y,z\right)\right),$$

Finally, the module uses a residual connection to obtain the output features:

(13) $$X_{out}=X_{bev}+0.5Y.$$

Through the above process, 2DMamba models global spatial dependencies within the BEV plane. Combined with the aforementioned Z-to-Channel cross-dimensional reorganization strategy, voxel features are transformed into a BEV-compatible representation, enabling 2DMamba to perform global spatial modeling on the BEV plane.

#### 3.2.3. Convolutional Feed-Forward Network and Inverse Reconstruction

To further fuse the BEV features after the 2DMamba scan, we introduce a Convolutional Feed-Forward Network (ConvFFN) following the 2DMamba block. Specifically, ConvFFN consists of a 1 × 1 convolution, a ReLU activation, a 3 × 3 convolution, another ReLU activation, and a final 1 × 1 convolution. All three convolutional layers use a stride of 1 and preserve the input channel dimension, while the 3 × 3 convolution uses a padding size of 1 to maintain the spatial resolution. These lightweight convolutional layers and nonlinear activation functions are used to enhance the representation of local spatial details and form a local-global complementarity with the global dependency modeling of 2DMamba. Subsequently, the model performs a Channel-to-Z inverse reorganization operation that is symmetric to the Z-to-Channel operation, restoring the BEV features.

(14) $$Y_{bev}\in R^{B\times H\times W\times (ZC)},$$

back into a five-dimensional voxel feature representation that includes the height dimension:

(15) $$Y_{voxel}\in R^{B\times H\times W\times Z\times C}.$$

Finally, based on the VPM module proposed in Section 3.1.3, the model utilizes voxel coordinate indices and the voxel-to-point reverse mapping relationship to inject the enhanced voxel features back into their corresponding original points. Through the aforementioned alternating modeling process of point–voxel–BEV–voxel–point, Mamba-BEV models global spatial dependencies in the BEV plane.

### 3.3. Multiscale Voxel-Point Alternating Fusion (MVPF) Module

We introduce an alternating multiscale point–voxel hierarchy to represent objects at different scales while reducing cross-scale spatial misalignment. This structure comprises four consecutive levels, corresponding to the base voxel scales: 1×, 2×, 4×, and  8×. During the multiscale modeling process, the voxel size is incrementally increased only in the X and Y planes, while the voxel partitioning in the height direction remains unchanged, thereby expanding the BEV receptive field.

**Figure 3:**
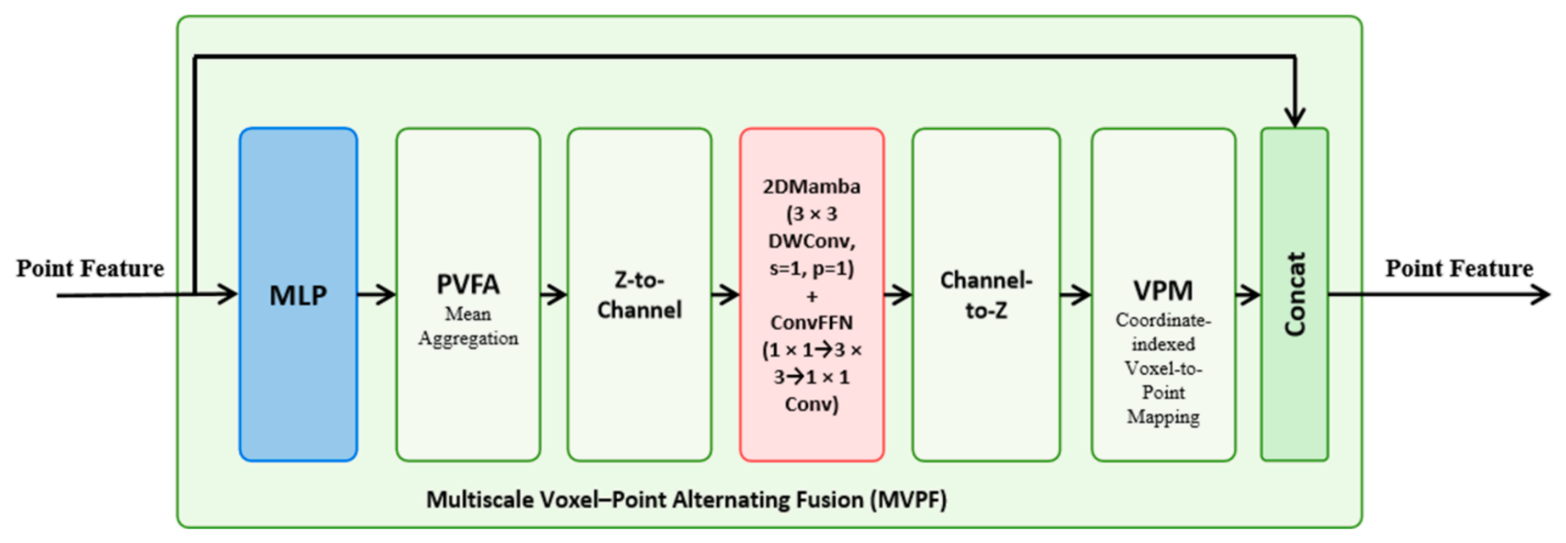
Architecture of the Single-Scale Voxel–Point Alternating Fusion Module. At scale s, point features are aggregated into voxel features through PVFA and reorganized into BEV features by Z-to-Channel. After global spatial modeling and local feature refinement through 2DMamba and ConvFFN, respectively, Channel-to-Z restores the voxel representation, and VPM maps the enhanced voxel features back to the corresponding points. The enhanced point features are then concatenated with the current input point features and propagated to the next scale.

Unlike methods that rely on continuous downsampling of high-resolution voxel features, we regenerate voxel indices at each scale based on the original point cloud coordinates. The voxel grids at each scale are directly partitioned based on the points’ actual spatial positions, allowing point features to be propagated across different scales while maintaining geometric consistency. This approach mitigates issues such as cross-resolution grid misalignment and feature mixing between scales. The architecture of a single-scale MVPF stage is illustrated in Figure 3.

The four MVPF levels take point features with dimensions of 16, 32, 64, and 128 as input, respectively. Before voxel aggregation, a scale-specific MLP expands the point-feature dimension by a factor of eight, producing voxel-feature dimensions of 128, 256, 512, and 1024. At the s-th level, the resulting voxel-feature dimension is denoted by $C_{s}$. Each level contains one 2DMamba block whose model dimension is determined by the Z-to-Channel output, namely $d_{model}=ZC_{s}$. The expansion factor is set to 2, the state dimension is fixed to 8, and the time-step rank is automatically determined as ${[d}_{model}/16]$.

Specifically, voxel indices are regenerated from the original point coordinates according to the voxel size of each level. The current point features are first aggregated into sparse voxel features through the PVFA module. These sparse features are then placed into a regular five-dimensional tensor according to their voxel coordinates. At the s-th level, the voxel tensor has the shape $\;[B,H_{s},W_{s},Z,C_{s}]$ where B is the batch size and $H_{s}$, $W_{s}$, and Z denote the voxel-grid dimensions. Z-to-Channel subsequently merges the height and channel dimensions and transforms the voxel tensor into $\left[B,H_{s},W_{s},ZC_{s}\right]$, producing the BEV feature at the current scale:

(16) $$X_{bev}^{\left(s\right)}\in R^{B\times H_{s}\times W_{s}\times \left(ZC_{s}\right)},s=1,2,4,8$$

The reorganized features are fed into 2DMamba to model global spatial dependencies within the BEV plane. Subsequently, ConvFFN further refines the local spatial information while preserving the feature-channel dimension and spatial resolution. After global and local feature modeling, Channel-to-Z restores the five-dimensional voxel representation, and VPM re-injects the enhanced voxel features into their corresponding original points. The enhanced point features at each level are concatenated with the current input features along the channel dimension and progressively propagated to the next level, enabling the point-level representation to continuously integrate multiscale contextual information. The input point-feature dimensions of the four MVPF levels are 16, 32, 64, and 128, respectively, and the corresponding output dimensions after feature concatenation are 32, 64, 128, and 256. The fine-scale levels focus on preserving local spatial structures, whereas the coarse-scale levels provide larger BEV receptive fields and enhance large-scale semantic modeling. Since the voxel indices are regenerated from the original point coordinates at every level, the point features remain geometrically aligned during cross-scale propagation. We further introduce Fourier positional encoding to provide positional information during repeated point–voxel feature transformations. Through this hierarchical alternating process, MVPF integrates point-level geometric information, BEV global dependencies, and multiscale semantic context, while point-feature propagation across scales helps reduce spatial misalignment.

After four levels of alternating point–voxel modeling, the resulting 256-dimensional point features are processed by a three-layer post-processing MLP. The first layer maps the feature dimension from 256 to 128, while the subsequent two layers maintain the 128-dimensional representation. The final 128-dimensional point features are aggregated using scatter-max according to the voxel indices at the finest level, generating sparse voxel features and their corresponding coordinates for the subsequent BEV backbone and detection head.

## 4. Experiments

### 4.1. Experimental Setup

This section presents the experimental setup, quantitative performance comparisons, ablation studies, and qualitative visualization analysis of Mamba-BEV. For the models reproduced in this study, all experiments were conducted under the same hardware and software environment. Specifically, we fully reproduced SECOND, PointPillars, and the VoxSeT baseline using two NVIDIA GeForce RTX 3090 GPUs (NVIDIA Corporation, Santa Clara, CA, USA), while the 3D detection results of the other comparison methods were obtained from the publicly reported benchmark results in their respective original publications. In addition, the inference speeds of our method and the baseline model were evaluated under the same experimental configuration.

#### 4.1.1. Dataset

This paper uses the KITTI 3D object detection benchmark dataset for evaluation. The dataset contains 7481 training samples and 7518 test samples, covering three core object categories: Car, Pedestrian, and Cyclist. KITTI defines Easy, Moderate, and Hard subsets according to minimum image-space bounding-box height, occlusion level, and truncation ratio. Following the industry-standard division, the training set is further split into 3712 training samples and 3769 validation samples. All models are trained on the training subset and evaluated on the validation subset.

#### 4.1.2. Evaluation Metrics

This paper adopts the official KITTI 3D object detection evaluation protocol to evaluate three object classes: Car, Pedestrian, and Cyclist. The evaluation metrics are 3D Average Precision (3D AP) and 3D mean Average Precision (3D mAP), and all results are reported under the Easy, Moderate, and Hard difficulty levels defined by the KITTI benchmark. The IoU threshold is set to 0.7 for the Car class and 0.5 for the Pedestrian and Cyclist classes.

The VoxSeT baseline adopted in this paper and the publicly reported results of some mainstream comparison methods are all based on AP_R11_. Therefore, all experiments in this paper are consistently evaluated using AP_R11_ on the KITTI validation set. The main experiments evaluate the overall performance of the proposed method against mainstream detectors, while the ablation experiments analyze the independent contributions of each module under the Moderate difficulty level. To further assess the robustness of the reported results, bootstrap resampling was performed on the prediction results of the 3769 validation samples with replacement for 1000 iterations, and the results are reported as the mean ± standard deviation.

#### 4.1.3. Experimental Environment and Training Details

The experimental server is equipped with two NVIDIA GeForce RTX 3090 GPUs (24 GB of VRAM per GPU; NVIDIA Corporation, Santa Clara, CA, USA) and an Intel Core i9-10900K CPU (Intel Corporation, Santa Clara, CA, USA). The software environment is Ubuntu 20.04 LTS (Canonical Group Limited, London, UK); the deep learning framework is PyTorch 1.8.1 (Facebook AI Research, Menlo Park, CA, USA), and the CUDA version is 11.1 (NVIDIA Corporation, Santa Clara, CA, USA). During training, the batch size per GPU was set to 4, resulting in a total batch size of 8, and the model was trained for a total of 100 epochs.

The input point cloud ranges were set to x∈[0, 69.12], y∈[−39.68, 39.68], and z∈[−3, 1]. The base voxel size was set to 0.32 m×0.32 m×4 m, and the maximum number of voxels for the training and testing phases was set to 16,000 and 40,000, respectively. During data preprocessing, points and bounding boxes outside the point cloud ranges were first removed; subsequently, during the training phase, the point order was randomly shuffled, and the point cloud was converted to a voxel representation.

The input and output feature dimensions of the VFE were set to 16 and 128, respectively. The four MVPF levels used point-feature dimensions of 16, 32, 64, and 128, while the corresponding channel dimensions used by 2DMamba and ConvFFN were 128, 256, 512, and 1024. Each level contained one 2DMamba block. With an expansion factor of 2, the corresponding inner dimensions were 256, 512, 1024, and 2048, while the time-step ranks were set to 8, 16, 32, and 64, respectively. The ConvFFN feature tensors at the four levels had the shapes [B, 128, 216, 248], [B, 256, 108, 124], [B, 512, 54, 62], and [B, 1024, 27, 31], respectively. After multiscale point–voxel processing, the resulting sparse voxel features were projected onto the BEV plane and further processed by the two-stage BEV backbone before being fed into the detection head.

The training phase employs data augmentation strategies consistent with the VoxSeT baseline, including ground-truth sampling, random flipping along the x axes, random global rotation, and random global scaling. Specifically, the random rotation range is $\left[-0.785,\;0.785\right]$, and the random scaling range is [0.95, 1.05]. Random data augmentation is not used during the validation and inference phases.

The optimizer uses the Adam OneCycle strategy, with an initial learning rate of 0.0015, weight decay set to 0.01, a momentum parameter of 0.9, a OneCycle momentum range of [0.95, 0.85], and pct_start set to 0.4. Gradient clipping is applied during training, with the maximum gradient norm set to 10. Except for the proposed architectural changes, the baseline and Mamba-BEV use the same data split, input range, augmentation, training schedule, and post-processing settings.

### 4.2. Main Experimental Results and Analysis

#### 4.2.1. Detection Accuracy Comparison

Table 1 shows a comparison of the 3D AP_R11_ performance between Mamba-BEV and mainstream 3D object detection methods on the KITTI validation set. Methods marked with “*” indicate results reproduced in this study under identical experimental conditions to ensure a fair comparison, whereas unmarked results are directly cited from the corresponding original publications.

As shown in Table 1, Mamba-BEV achieved better detection performance than the baseline VoxSeT for all three target categories: Car, Pedestrian, and Cyclist. Under the Moderate difficulty level, Mamba-BEV achieved 3D AP values of 78.8%, 55.4%, and 68.0% for the Car, Pedestrian, and Cyclist categories, respectively, representing improvements of 0.6%, 1.5%, and 0.4% over the baseline model. The improvement is most pronounced for the Pedestrian class, with a 2.4% gain under the Hard difficulty level, indicating that our method achieves better detection performance than the baseline model for sparse and small objects. Compared with WinMamba, Mamba-BEV achieves higher Car 3D AP under all three difficulty levels, whereas WinMamba shows better performance in the Pedestrian and Cyclist categories.

The category-wise results can also be considered in relation to the characteristics of different object classes. Car targets generally have larger physical dimensions and denser point distributions, and most detectors already achieve relatively high detection accuracy for this class. In contrast, Pedestrian and Cyclist targets are typically smaller, more sparsely sampled, and more frequently affected by occlusion, making their detection more challenging. In Mamba-BEV, Z-to-Channel reorganizes voxel features into a BEV-compatible representation and works with 2DMamba to model spatial dependencies within the BEV plane. Meanwhile, LVFE re-injects the enhanced voxel features into their corresponding original points through coordinate-indexed mapping to preserve point–voxel spatial correspondence, while the multiscale voxel–point alternating fusion further helps reduce spatial misalignment during cross-scale feature propagation. Together, these designs improve the modeling of different object categories, thereby contributing to better overall detection performance.

#### 4.2.2. Inference Efficiency Comparison

Under the same experimental conditions, we further compare the computational efficiency of the baseline and different architectural configurations, as shown in Table 2.

As shown in Table 2, the configurations containing MVPF have higher parameter counts and GFLOPs, indicating that they require more floating-point operations, yet their actual inference speed remains higher than that of the baseline model. This suggests that GFLOPs mainly reflect the theoretical floating-point computational cost, whereas practical execution efficiency is also jointly affected by factors such as operator implementation, memory-access patterns, and GPU resource utilization. Therefore, computational cost and actual inference time do not exhibit a simple linear relationship.

MVPF performs feature modeling on regular multiscale BEV tensors and employs the compiled 2DMamba CUDA operator for 2D selective scanning. The hardware-aware scan of 2DMamba conducts horizontal and vertical information aggregation through 2D tiling and on-chip caching, thereby reducing the explicit storage of intermediate states and memory transfers while improving GPU execution efficiency. Consequently, although MVPF increases the overall number of floating-point operations, the CUDA-optimized 2DMamba operator still enables higher practical inference efficiency.

The results of the different configurations further clarify the efficiency contribution of each component. Adding LVFE alone to the baseline introduces additional local sparse-voxel enhancement and point–voxel mapping operations, resulting in a certain amount of runtime overhead. In contrast, replacing the original VSA module with MVPF improves inference efficiency. Therefore, the speed improvement of Mamba-BEV mainly benefits from the CUDA-optimized 2DMamba operator within MVPF, whereas LVFE is primarily designed to enhance local voxel representations and introduces only a small amount of additional computational overhead.

### 4.3. Ablation Experiments

To validate the effectiveness of each module in Mamba-BEV, we conduct system-level ablation experiments on the KITTI validation set. All ablation experiments were conducted under the same training configuration, hardware environment, and evaluation protocol, using 3D AP_R11_ at the Moderate difficulty level as the evaluation metric.

#### 4.3.1. Ablation Experiments on Different Architectural Configurations

To analyze the contributions of the main architectural components to the overall detection performance, we conduct combination experiments on positional encoding (PE), the Local Voxel Feature Enhancement (LVFE) module, and the Multiscale Voxel–Point Alternating Fusion (MVPF) module. The results are shown in Table 3.

As shown in Table 3, the baseline model achieves an overall 3D mAP of 66.58%. When PE and LVFE are introduced without MVPF, the overall mAP increases to 67.21%, with improvements of 0.45% and 1.74% in the Car and Pedestrian categories, respectively. After MVPF is further incorporated to form the complete model, the overall mAP increases to 67.45%. In particular, the Cyclist AP increases from 67.32% to 68.02%, indicating that the multiscale point–voxel alternating fusion contributes to feature modeling across different voxel scales.

When PE and MVPF are retained without LVFE, the model also achieves an overall mAP of 67.45%. Incorporating LVFE further improves the Car and Pedestrian AP from 78.15% and 54.51% to 78.85% and 55.49%, respectively, although the Cyclist AP decreases from 69.69% to 68.02%. Consequently, the overall mAP remains unchanged. This result indicates that LVFE mainly improves the local voxel representations of the Car and Pedestrian categories, while its performance gains vary across different object classes. The specific contribution of LVFE is further analyzed in Section 4.3.2.

In contrast, when PE is removed while LVFE and MVPF are retained, the overall mAP decreases to 62.24%, with substantial performance degradation in the Pedestrian and Cyclist categories. Reintroducing PE increases the overall mAP by 5.21% and improves the detection performance of all three categories. This demonstrates that PE is important for preserving relative positional information during repeated voxelization and point–voxel feature propagation. With PE, LVFE, and MVPF jointly enabled, the complete model achieves AP values of 78.85%, 55.49%, and 68.02% for Car, Pedestrian, and Cyclist, respectively, resulting in an overall mAP of 67.45%, which is 0.87% higher than that of the baseline. Overall, PE provides essential positional information, while LVFE and MVPF contribute to local voxel enhancement and multiscale feature modeling, respectively.

#### 4.3.2. Ablation Study of the LVFE Module

To evaluate the effectiveness of the proposed LVFE module and investigate the influence of different voxel densification designs, we compare three configurations: without LVFE, LVFE with the full VDM, and LVFE with the lightweight VDM. In the configuration without LVFE, both the VDM and VPM components within LVFE are removed. The results are shown in Table 4.

Experimental results show that, without LVFE, the model achieves an overall mAP of 67.45% under the Moderate difficulty level. When LVFE is implemented with the full VDM, the model performance decreases to 65.27%. This suggests that excessively deep 3D sparse convolutional stacking may introduce redundant neighborhood information, thereby weakening the model’s discriminative capability for small objects. In contrast, LVFE with the lightweight VDM also achieves an overall mAP of 67.45% under the Moderate difficulty level. However, when all object categories and difficulty levels are considered, its overall mAP reaches 70.02%, which is higher than the 69.50% obtained without LVFE. This configuration moderately enhances local voxel representations and re-injects the enhanced voxel features into the original points without introducing excessive computational complexity, resulting in more balanced detection performance across different categories and difficulty levels.

#### 4.3.3. Ablation Study of the Voxel-to-Point Mapping (VPM) Module

To validate the effectiveness of the proposed Voxel-to-Point Mapping (VPM) module, we keep the VDM and all other network configurations unchanged and replace only the voxel-to-point feature mapping strategy. We compare three mapping methods: nearest-neighbor interpolation, bilinear interpolation, and the proposed VPM. The results are shown in Table 5.

Experimental results show that nearest-neighbor interpolation and bilinear interpolation achieve overall mAP values of 65.90% and 65.91%, respectively. In contrast, the proposed VPM improves the overall mAP to 67.45% and achieves better detection performance across all three object classes. This indicates that, compared with interpolation-based feature propagation methods, directly assigning voxel features according to the recorded point–voxel correspondence can better maintain the correspondence between each point and its associated voxel while reducing cross-voxel feature mixing.

#### 4.3.4. Ablation Study on BEV Feature Transformation Strategies

To analyze the impact of different BEV transformation strategies on detection performance, we compare four strategies: traditional height-axis max pooling, traditional height-axis average pooling, Z-to-Channel cross-dimensional reorganization, and learnable *Z*-axis convolutional compression. The results are shown in Table 6.

Experimental results show that max-pooling, average-pooling, and learnable *Z*-axis convolution achieve overall mAP values of 67.16%, 66.50%, and 66.29%, respectively. In contrast, the proposed Z-to-Channel strategy achieves the highest overall mAP of 67.45% and demonstrates good detection performance across all three object classes. In addition, Z-to-Channel introduces no additional learnable parameters while maintaining an overall computational cost comparable to those of the other transformation strategies. By reorganizing the Z dimension into the channel dimension, Z-to-Channel forms a symmetric transformation with Channel-to-Z, which restores the processed BEV features to the voxel representation for subsequent voxel-to-point feature re-injection. Therefore, Z-to-Channel is adopted because it achieves better detection performance at a comparable computational cost while being more suitable for the proposed voxel–BEV–point alternating processing framework.

#### 4.3.5. Ablation Study on Vertical Voxel Partitioning

To investigate the influence of vertical voxel discretization, we compare the default configuration with (Z = 1) and a finer vertical discretization with (Z = 4), while keeping the other experimental settings unchanged. The results are shown in Table 7.

The Z = 1 configuration achieves higher detection performance across all three object categories, with an overall mAP of 67.45%, compared with 65.94% for Z = 4, while also requiring less training time. These results indicate that finer vertical voxel discretization does not further improve detection performance under the current framework and increases the training cost. Therefore, Z = 1 is adopted as the default configuration in the main experiments.

#### 4.3.6. Multiscale Processing Mechanism Ablation

To verify the effectiveness of the alternating propagation mechanism between multiscale voxels and point features, we compare it with a pure voxel-based multiscale propagation strategy.

As shown in Table 8, the overall mAP of pure voxel-based multiscale propagation is 66.51%, whereas the proposed point–voxel alternating propagation mechanism increases the overall mAP to 67.45%. This result indicates that alternating point and voxel representations during multiscale feature propagation can achieve better overall detection performance than using voxel representations alone.

Repeated downsampling in pure voxel-based multiscale propagation may affect cross-scale feature consistency. In contrast, our method regenerates voxel indices at each scale based on the original point cloud coordinates and performs voxel aggregation, 2DMamba modeling, and point feature re-injection at each level. Point features are propagated across different scales to help maintain spatial correspondence during cross-scale feature propagation. Through this alternating voxel–point propagation process, multiscale information can be progressively integrated while preserving the coordinate correspondence between point and voxel features.

#### 4.3.7. Ablation Study on the Number of Voxel Scales

To investigate the influence of the number of voxel scales in the proposed MVPF module, we construct four model variants containing one, two, three, and four voxel scales, respectively. All other modules and experimental settings are kept unchanged, and only the number of MVPF levels is varied. The results are presented in Table 9.

Although the three-scale configuration achieves a slightly higher category-averaged 3D mAP under the Moderate difficulty level, the four-scale configuration achieves a higher overall mAP when all object categories and difficulty levels are considered, increasing from 69.89% to 70.02%. In addition, the Car 3D AP under the Easy difficulty level increases from 87.77% to 89.00%. Therefore, the four-scale configuration is adopted in the final model.

### 4.4. Qualitative Visualization Analysis

To further intuitively validate the detection performance of Mamba-BEV in complex road scenes, we conduct a qualitative visualization analysis based on the KITTI validation set. In the experiments, multiple typical scenes were randomly selected to compare the original images, the detection results of the baseline model, and the detection results of the proposed model. In the figure, the red circles highlight the main areas of difference in the detection results between the two methods.

**Figure 4:**
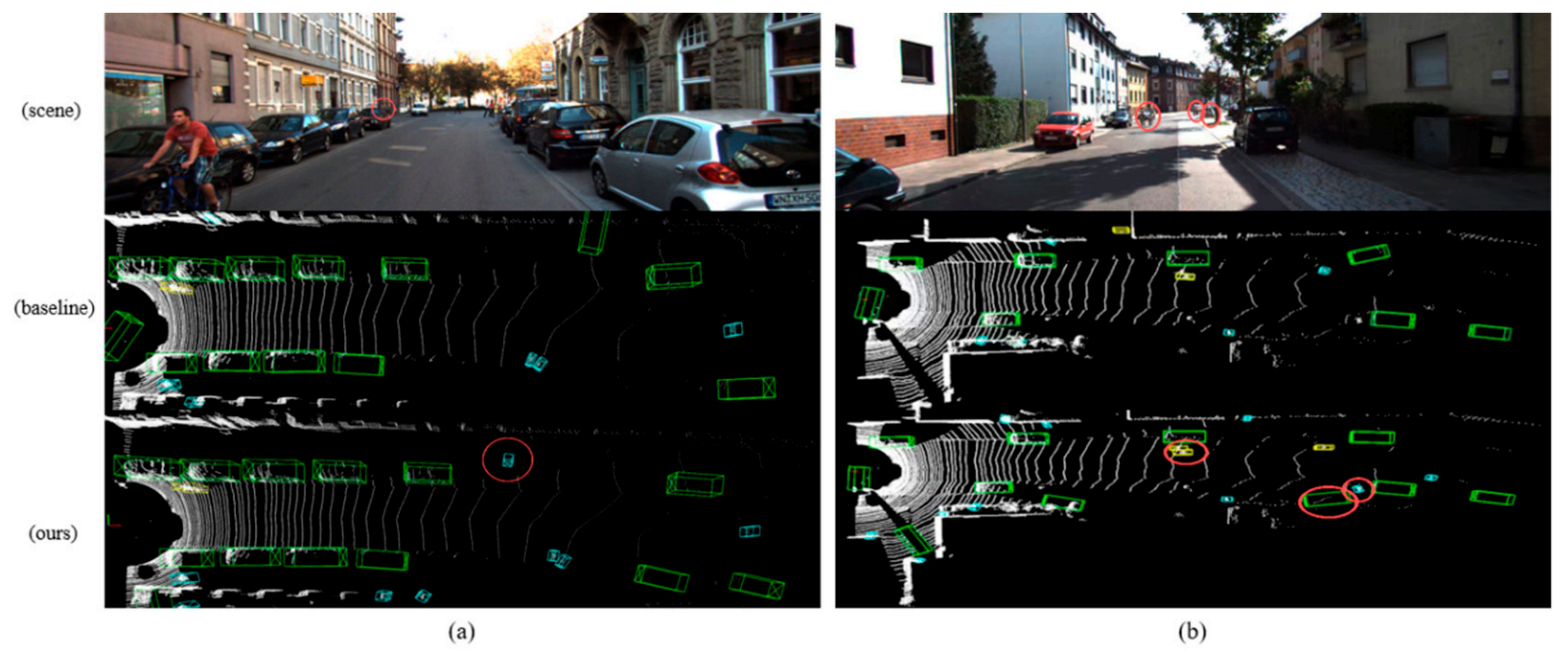
Visual comparison of the detection results for distant small objects and occluded objects. Specifically, (**a**) displays the detection results for small distant targets in the same scene, while (**b**) shows the detection results for distant occluded targets in the same scene. Green, blue, and yellow bounding boxes represent Cars, Pedestrians, and Cyclists, respectively. The red circles highlight the main areas of difference between the baseline model and the method proposed in this paper.

**Figure 5:**
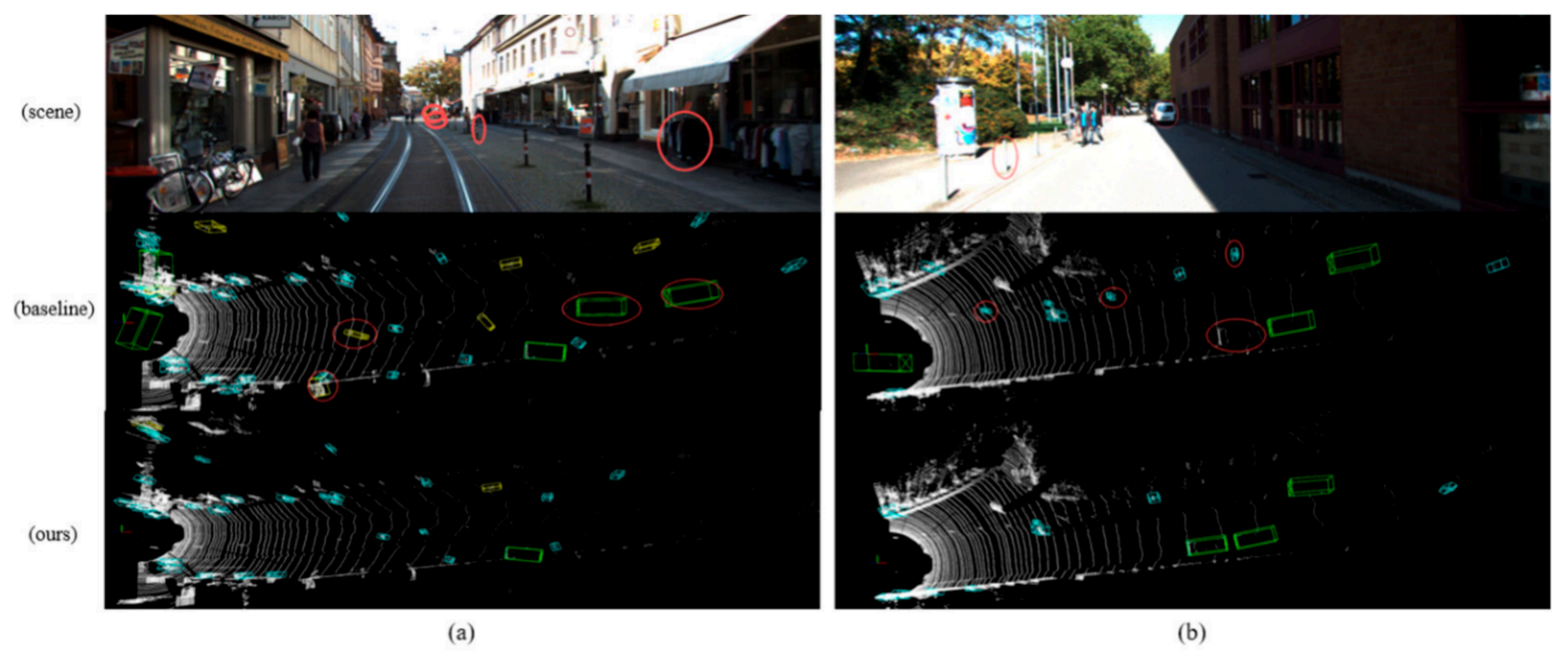
Detection results showing false positives and false negatives in two different scenarios. Specifically, (**a**,**b**) show detection results in two different road scenes. Green, blue, and yellow bounding boxes represent Cars, Pedestrians, and Cyclists, respectively. The red circles highlight the main areas of difference between the baseline model and the method proposed in this paper.

As shown in Figure 4, in the selected scenes, Mamba-BEV detects several distant pedestrians, cyclists, and partially occluded objects that are missed by the baseline model. Figure 5 further presents examples containing false-positive and false-negative predictions. In these specific cases, compared with the baseline model, Mamba-BEV produces fewer missed detections for some distant objects and reduces certain incorrect predictions in complex background regions. These results provide an intuitive illustration of the detection performance of Mamba-BEV in the selected scenes.

## 5. Conclusions

This paper proposed Mamba-BEV, a single-stage state-space framework for LiDAR point cloud-based 3D object detection. Two modules were designed in this work, namely, the Local Voxel Feature Enhancement (LVFE) module and Multiscale Voxel–Point Alternating Fusion (MVPF) module. LVFE integrated Point-to-Voxel Feature Aggregation (PVFA), Voxel Densification (VDM), and coordinate-indexed Voxel-to-Point Mapping (VPM) to enhance local voxel representations while maintaining point–voxel spatial correspondence. MVPF combined Z-to-Channel cross-dimensional reorganization with 2DMamba-based global spatial modeling at multiple voxel scales, while propagating point features across different scales to maintain cross-scale geometric consistency. By integrating these two modules, Mamba-BEV unifies local voxel feature enhancement, global BEV spatial modeling, and multiscale point–voxel feature propagation within a single framework. Experimental results on the KITTI validation set show that, compared with the VoxSeT baseline, Mamba-BEV could achieve higher detection accuracy across all three object categories and a higher inference speed under the same experimental conditions. For practical autonomous driving deployment, further consideration should be given to memory overhead, inference latency stability, hardware compatibility, multiple dataset evaluation and detection performance under diverse environmental conditions. Future work will focus on developing more lightweight state-space modeling and multiscale feature fusion strategies to further reduce computational overhead while maintaining effective local and global feature modeling.

## Figures and Tables

**Table 1:** Comparison of 3D Object Detection Performance on the KITTI Validation Set.

Model	Reference	Car. 3D (AP_R11_)			Ped. 3D (AP_R11_)			Cyc. 3D (AP_R11_)		
		Easy	Mod.	Hard	Easy	Mod	Hard	Easy	Mod	Hard
SECOND * [6]	Sensors 2018	88.1	78.3	77.1	57.3	53.0	48.8	80.0	64.9	61.6
VoxelNet [5]	CVPR 2018	87.8	75.5	72.7	56.4	50.9	45.6	78.1	61.7	54.6
PointPillars * [16]	CVPR 2019	85.5	77.0	75.2	56.5	51.1	47.1	81.0	63.5	59.8
TANet [36]	AAAI 2020	83.8	75.3	67.6	54.9	46.6	42.4	73.8	59.8	53.4
PillarNet [37]	ECCV 2022	87.3	77.7	76.6	47.7	43.8	41.3	80.0	60.9	57.9
DSVT-Pillar [26]	CVPR 2023	87.3	77.4	76.2	61.4	56.8	51.8	82.3	67.1	63.7
DSVT-Voxel [26]	CVPR 2023	87.8	77.8	76.8	66.1	59.7	55.2	83.5	66.7	63.2
LION-Mamba [33]	NeurIPS 2024	88.6	78.3	77.2	67.2	60.2	55.6	83.0	68.6	63.9
WinMamba [34]	AAAI 2026	88.4	78.2	77.1	**69.3**	**63.3**	**57.7**	**90.1**	**71.4**	**67.3**
VoxSeT * [10]	CVPR 2022	88.6	78.2	77.0	57.7	53.9	48.6	83.9	67.6	64.7
**Ours**		**89.0**	**78.8**	**77.6**	59.5	55.4	51.0	85.6	68.0	64.9
Bootstrap mean ± standard deviation		89.0 ±0.2	78.9 ±0.3	77.7 ±0.2	59.7 ±1.6	55.4 ±1.4	50.9 ±1.4	85.6 ±1.6	68.0 ±2.0	64.7 ±2.0

Note: Bold values indicate the best performance in each category.

**Table 2:** Comparison of Inference Efficiency Among Different Architectural Configurations.

Architecture Configuration		Params (M)	GFLOPs	Latency (ms)	FPS
LVFE	MVPF				
Baseline		3.022	102.199	22.7	44.05
√		3.029	102.199	27.1	36.90
	√	25.773	203.454	14.6	68.49
√	√	25.776	203.454	14.9	67.11

Note: LVFE is directly added to the baseline as a local voxel feature enhancement module. When MVPF is introduced, it replaces the original VSA module in VoxSeT. The configuration incorporating both MVPF and LVFE represents the complete Mamba-BEV model. All configurations were evaluated under the same experimental settings, with the inference batch size set to 8. The reported latency is the average per-sample latency. The timing includes model inference and post-processing but excludes the subsequent KITTI AP evaluation. FPS is calculated as the reciprocal of the Latency. √ indicates that the corresponding module is added.

**Table 3:** Ablation Results of Different Architectural Configurations.

Architecture Configuration			Car. 3D (AP_R11_)	Ped. 3D (AP_R11_)	Cyc. 3D (AP_R11_)	3D mAP_R11_
PE	LVFE	MVPF				
Baseline			78.23	53.90	67.62	66.58
√	√		78.68	55.64	67.32	67.21
√		√	78.15	54.51	69.69	67.45
	√	√	77.71	47.55	61.46	62.24
√	√	√	78.85	55.49	68.02	67.45

Note: √ indicates that the corresponding module is added.

**Table 4:** Ablation Results for the VDM Component of LVFE.

Architectural Configuration	Car. 3D (AP_R11_)	Ped. 3D (AP_R11_)	Cyc. 3D (AP_R11_)	3D mAP_R11_
Without LVFE	78.15	54.51	69.69	67.45
LVFE with Full VDM	78.14	53.30	64.38	65.27
LVFE with Lightweight VDM	78.85	55.49	68.02	67.45

Note: To facilitate the subsequent overall performance evaluation, the AP_R11_ results without LVFE under the Easy, Moderate, and Hard difficulty levels are 88.18, 78.15, and 77.00 for Car; 57.78, 54.51, and 49.58 for Pedestrian; and 86.03, 69.69, and 64.57 for Cyclist, respectively. For LVFE with the lightweight VDM, the corresponding results are 89.00, 78.85, and 77.63 for Car; 59.56, 55.49, and 51.08 for Pedestrian; and 85.60, 68.02, and 64.96 for Cyclist, respectively.

**Table 5:** Ablation Experiment Results for Different Voxel-to-Point Feature Mapping Methods.

Architecture Configuration	Car. 3D (AP_R11_)	Ped. 3D (AP_R11_)	Cyc. 3D (AP_R11_)	3D mAP_R11_
Nearest-Neighbor Interpolation	78.18	53.93	65.58	65.90
Bilinear Interpolation	78.54	54.13	65.05	65.91
VPM (Ours)	78.85	55.49	68.02	67.45

**Table 6:** Ablation Experiment Results for Different BEV Feature Transformation Strategies.

BEV Feature Transformation Strategies	Car. 3D (AP_R11_)	Ped. 3D (AP_R11_)	Cyc. 3D (AP_R11_)	3D mAP_R11_	GFLOPs	Params (M)
Max-Pooling-Based Transformation	78.22	55.35	67.91	67.16	203.454	25.773
Average-Pooling-Based Transformation	78.43	55.41	65.66	66.50	203.454	25.773
Learnable *Z*-axis Convolutional Transformation	78.24	54.66	65.96	66.29	203.454	27.16
Z-to-Channel (Ours)	78.85	55.49	68.02	67.45	203.454	25.773

**Table 7:** Ablation results for different vertical voxel partitioning settings.

*Z*-Axis Voxel Bins	Car. 3D (AP_R11_)	Ped. 3D (AP_R11_)	Cyc. 3D (AP_R11_)	3D mAP_R11_
Z = 1	78.85	55.49	68.02	67.45
Z = 4	78.26	54.16	65.41	65.94

**Table 8:** Results of the Multiscale Feature Propagation Mechanism Ablation Experiment.

Cross-Scale Feature Propagation Mode	Car. 3D (AP_R11_)	Ped. 3D (AP_R11_)	Cyc. 3D (AP_R11_)	3D mAP_R11_
Pure Voxel-Based Multiscale Propagation	78.35	54.03	67.15	66.51
Multiscale Point-Voxel Alternating Propagation	78.85	55.49	68.02	67.45

**Table 9:** Ablation Results for Different Numbers of Voxel Scales.

Number of Voxel Scales	Scale Factors	Car. 3D (AP_R11_)	Ped. 3D (AP_R11_)	Cyc. 3D (AP_R11_)	3D mAP_R11_
1	1	77.50	51.58	64.78	64.62
2	1, 2	77.76	56.64	64.73	66.38
3	1, 2, 4	78.14	55.57	68.79	67.50
4	1, 2, 4, 8	78.85	55.49	68.02	67.45

Note: To facilitate the overall performance comparison, the AP_R11_ results of the three-scale configuration under the Easy, Moderate, and Hard difficulty levels are 87.77, 78.14, and 76.95 for Car; 60.34, 55.57, and 51.06 for Pedestrian; and 85.68, 68.79, and 64.68 for Cyclist, respectively. For the four-scale configuration, the corresponding results are 89.00, 78.85, and 77.63 for Car; 59.56, 55.49, and 51.08 for Pedestrian; and 85.60, 68.02, and 64.96 for Cyclist, respectively.

## Data Availability

The original data presented in the study are openly available in KITTI official website: https://www.cvlibs.net/datasets/kitti/ (accessed on 15 August 2025).

## Abbreviations

The following abbreviations are used in this manuscript:

3D	Three-Dimensional
2D	Two-Dimensional
AP	Average Precision
mAP	mean Average Precision
FPS	Frames per second
IoU	Intersection over union
SSM	State-Space Model
BEV	Bird’s-Eye View
VFE	Voxel Feature Encoding
VDM	Voxel Densification Module
VPM	Voxel-to-Point Mapping
PVFA	Point-to-Voxel Feature Aggregation
LVFE	Local Voxel Feature Enhancement
MVPF	Multiscale Voxel–Point Alternating Fusion
